# Hepatic fat is related to neurodegeneration in midlife obesity

**DOI:** 10.1002/alz.091562

**Published:** 2025-01-09

**Authors:** Sara Hosseinzadeh Kasani, Mahsa Dolatshahi, Mahshid Naghashzadeh, Paul K. Commean, Farzaneh Rahmani, Jingxia Liu, LaKisha Lloyd, Caitlyn Nguyen, Nancy Hantler, Abby McBee‐Kemper, Maria Ly, Gary Z Yu, Joseph E. Ippolito, Jake Weeks, Lael Ceriani, Claude Sirlin, John C. Morris, Tammie L.S. Benzinger, Cyrus A. Raji

**Affiliations:** ^1^ Mallinckrodt Institute of Radiology, Washington University in St. Louis, St. Louis, MO USA; ^2^ Washington University in St. Louis School of Medicine, St. Louis, MO USA; ^3^ Washington University in St. Louis, St. Louis, MO USA; ^4^ University of California, San Diego, La Jolla, CA USA; ^5^ Washington University in St. Louis, School of Medicine, St. Louis, MO USA; ^6^ Knight Alzheimer Disease Research Center, St. Louis, MO USA

## Abstract

**Background:**

Obesity in midlife, defined as body mass index (BMI) of 30 kg/m^2^ or higher in those between 40‐60 years, is related to higher Alzheimer’s disease (AD) later in life. Non‐alcoholic fatty liver disease, as a complication of obesity is associated with impaired cognitive function. We investigated the relationship between hepatic fat quantification by use of MRI‐derived Positron Density Fat Fraction (PDFF) and brain cortical thickness in cognitively normal midlife individuals.

**Method:**

Overall, 63 cognitively normal middle‐aged participants (Age: 50.46±6.19 years, female: 22 (71%), obesity: 32 (50.28 %), BMI: 36.46±4.9 kg/m^2^) underwent brain and abdominal 3T MRI. PDFF values were calculated using a trained U‐Net convolutional neural network (CNN) model to infer the hepatic PDFF maps from conventional T1‐weighted images. The CNN included the Adam optimizer for training with a learning rate of 1e‐4. Visceral and subcutaneous adipose tissue (VAT, SAT) were automatically segmented using VOXel Analysis Suite (Voxa). FreeSurfer 7.1.1 was used for automatic segmentation of cortical and subcortical brain regions using a probabilistic atlas, followed by visual inspection and if needed, manual editing. A multivariable linear regression analysis was carried out to test the association of PDFF, VAT, SAT, and BMI with brain cortical thickness, with age and sex as covariates.

**Result:**

We observed a statistically significant association between PDFF and VAT (p=3e‐7), BMI (p=2e‐4), and insulin resistance (1e‐4). Among late‐onset AD (LOAD) cortical regions, there was a significant inverse correlation (Adjusted R²=0.19, p<0.001) between left temporal pole thickness and PDFF. Similarly, higher VAT was associated with thinning in left temporal pole (Adjusted R²=0.22, p<0.001) and middle temporal lobe (Adjusted R²=0.12, p=0.014).

**Conclusion:**

Overall, our data suggest a potential role of hepatic fat fraction in promoting neurodegeneration in cognitively normal midlife individuals. These findings lend insight into a pathway that can be utilized for future AD risk reduction.

